# A Bridge between the Breath and the Brain: Synchronization of Respiration, a Pupillometric Marker of the Locus Coeruleus, and an EEG Marker of Attentional Control State

**DOI:** 10.3390/brainsci11101324

**Published:** 2021-10-06

**Authors:** Michael Christopher Melnychuk, Ian H. Robertson, Emanuele R. G. Plini, Paul M. Dockree

**Affiliations:** 1 Trinity College Dublin, D02 PN40 Dublin, Ireland; iroberts@tcd.ie (I.H.R.); pinie@tcd.ie (E.R.G.P.); dockreep@tcd.ie (P.M.D.); 2 Global Brain Health Institute, D02 PN40 Dublin, Ireland

**Keywords:** respiration, locus coeruleus, theta-beta ratio, eeg, coupling, synchronization, noradrenaline, pupil, breath

## Abstract

Yogic and meditative traditions have long held that the fluctuations of the breath and the mind are intimately related. While respiratory modulation of cortical activity and attentional switching are established, the extent to which electrophysiological markers of attention exhibit synchronization with respiration is unknown. To this end, we examined (1) frontal midline theta-beta ratio (TBR), an indicator of attentional control state known to correlate with mind wandering episodes and functional connectivity of the executive control network; (2) pupil diameter (PD), a known proxy measure of locus coeruleus (LC) noradrenergic activity; and (3) respiration for evidence of phase synchronization and information transfer (multivariate Granger causality) during quiet restful breathing. Our results indicate that both TBR and PD are simultaneously synchronized with the breath, suggesting an underlying oscillation of an attentionally relevant electrophysiological index that is phase-locked to the respiratory cycle which could have the potential to bias the attentional system into switching states. We highlight the LC’s pivotal role as a coupling mechanism between respiration and TBR, and elaborate on its dual functions as both a chemosensitive respiratory nucleus and a pacemaker of the attentional system. We further suggest that an appreciation of the dynamics of this weakly coupled oscillatory system could help deepen our understanding of the traditional claim of a relationship between breathing and attention.

## 1. Introduction

**Figure 1:**
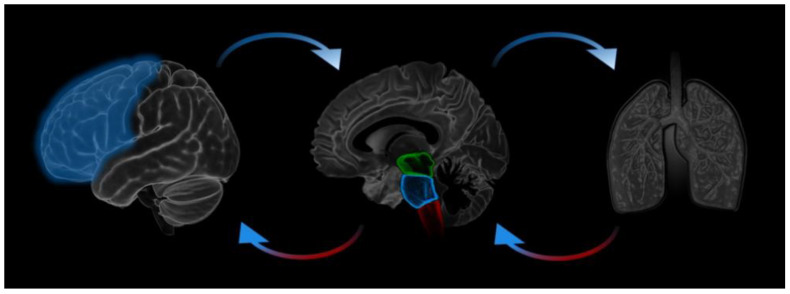
Three-way coupled oscillation of breathing, neurocognitive cortical processes and subcortical LC/NA modulation.

In this paper we investigate the extent to which key cortical and subcortical signatures of attention are synchronized with breath dynamics. To this end we examine a tripartite relationship between the respiratory cycle [1], the EEG theta-beta ratio [2], a putative marker of attentional control and mind wandering, and pupil diameter [3], a proxy measure for the locus coeruleus/noradrenergic system (Figure 1). The current aim is to understand bi-directional synchronization between these fundamental respiratory and attentional signals with a view to ultimately better understanding of how perturbation of the breath can affect attentional state and how this is modulated by the locus coeruleus (LC).

Yogic philosophy clearly states that the breath and the mind are closely related. This forms a foundational premise of many ancient techniques of breath control known collectively as *pranayama*. A primary goal of these practices is to stabilize the attention, or calm the *fluctuations of the mind* (*citta vritti* in Sanskrit), in order to prepare the mind for deep meditative practice. Yogic teachings suggest that the characteristics of the breath and the mental state are reflected in one another, and that by consistent observation and training of the breath, stability of attention and a tranquil mind can be achieved [4,5,6]. While it is currently agreed that at least some neuronal activity is regulated and/or entrained by respiratory activity [7,8,9,10,11,12,13], exactly how a relationship between the breath and “fluctuations of mind”, in a yogic context, might be understood from a neuroscientific perspective remains an open question.

Psychophysical experiments from the turn of the previous century revealed that attentional state and direct perceptual experience are both synchronized with the respiratory cycle [14,15,16,17,18,19]. Attentional state tends to switch, and the liminal threshold of perception similarly tend to wax and wane with the breath, suggesting the breathing cycle likely has subtle cognitive and behavioral consequences. These findings mate well with the yogic teachings concerning breath-related mental fluctuations, and suggest that there should be underlying electrophysiological evidence of this synchronization.

As mentioned, pranayama, or manipulation of the breath, prepares the mind for meditative practice by reducing the fluctuations of mind and attention that accompany normal day-to-day life, and facilitates achievement and maintenance of an unwavering single-pointed attention. Meditation, particularly when exclusively focused on the sensations of the breath, is a subtle attentional task with minimal exogenous motivation—the exigency for maintaining focus is almost entirely internally driven, and lapses in focus, particularly at the initial stages of practice, are extremely common. Mind wandering (MW) is said to have occurred when the focus falls away from the sensations of breathing, either briefly or for more prolonged periods, until the meditator becomes aware of the fact and returns their focus to their breath.

MW is, of course, a common feature of everyday experience as well, sometimes to our detriment. Some attempts have been made to elucidate the neural correlates and precursors of the MW process, of the disengagement and re-engagement of attention from and to the intended object or “task”. These studies generally employ “thought probes” to detect the occurrence of MW, and have focused, the most part, on task-related fMRI [20,21] and electrophysiological potentials such as the P300 [22,23]. There has been recent interest, however, in characterizing these lapses *spectrally* during focused attentional tasks [24,25,26], with delta, theta, alpha, and beta frequency bands (approx. *1–3 Hz, 3–7 Hz,* 7–13 Hz, 13–30 Hz, respectively) all varying with the occurrence of MW. Transient spectral correlates of MW specifically in the context of breath-focused meditation have also been observed [27], with an increase in low frequencies (delta and theta) and a decrease in higher frequencies (alpha and beta) observed during periods of MW. Interestingly, a recent study has found that the ratio of theta to beta frequencies (theta-beta ratio; TBR) increased during self-reported MW events and decreased following MW event-awareness [28]. This result is of particular interest, as TBR is an established marker of attentional control.

TBR, the ratio of slow (~3–7 Hz) to faster (~13–30 Hz) power in electroencephalographic (EEG) recordings, is considered an index of attentional control [29], and is thought to reflect the interactive tension between bottom-up emotionally driven subcortical networks, working memory and episodic memory retrieval (theta), and top-down goal-directed attentional processes (beta; [29,30,31]). TBR is higher in attention deficit hyperactivity disorder (ADHD; [32,33,34,35,36,37,38]), non-clinical populations self-reporting lower attentional control [30,39,40,41] and during experimentallyinduced reductions in attentional control [30]. Lower TBR is negatively associated with better emotional regulation [41], and higher TBR with increased risk taking [31,42,43] and poorer behavioral adaptation [44,45]. Resting state fluctuations of mind *without* a task and fluctuations of control *within* a task both exhibit the same directional association with TBR. That is, TBR provides an index of an attention state, whether cognitive processes are unconstrained internally or constrained by an external task.

As mentioned earlier, TBR at the frontal midline has recently been shown to also exhibit transient changes across relatively brief MW events during a breath-monitoring task [28] with TBR increasing during self-reported attentional lapses, and decreasing again following awareness. Functional connectivity (FC) of the executive control network (ECN; associated with goal-directed attentional control), was shown to increase with lower TBR in the same group of participants using fMRI [46], strongly suggesting TBR as an index of goal-directed attention on transient time scales. These findings suggest that not only does TBR provide a useful temporal index of MW and MW awareness, but also the extent to which nodes of the ECN are coordinated in neural signatures typical of focused attentional states. Their findings are perhaps expected, as ECN FC is known to decrease during MW events while meditating [47], but it is worth noting that van Son’s findings show TBR to index these network-level changes in real time.

Given the long-purported yogic teachings on the breath and fluctuations of the mind, the early evidence of respiratory-perceptual synchronization, along with the more recent imaging evidence of cortical and subcortical coupling with respiration, we sought here to investigate whether an electrophysiological index associated with attentional state and stability might show signal variability across the respiratory cycle. Following on from van Son’s findings of TBR signal changes during MW, we assessed here whether TBR would also show evidence of coupling with the cycle of the breath, oscillating in synchrony with it.

**Figure 2:**
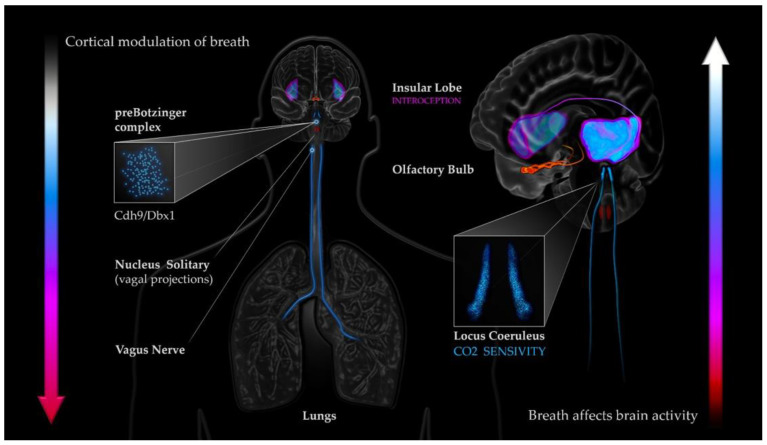
Potential coupling mechanisms between the breath and cortical activity.

Coupling between the breath and the mind obviously requires a nexus through which information/signal can be shared between systems, and which is amenable to change. It is likely there are multiple parallel pathways and mechanisms through which this might occur (Figure 2). The olfactory bulbs [1,48,49,50,51], the LC [9], subpopulations of brainstem (Cdh9/Dbx1) neurons [12], and stretch-receptor induced vagal inhibition [52] have all been hypothesized as potential respiratory-related modulators of cortical activity. Interoceptive information from respiratory behavior could also be involved, particularly in the case of breath-focused meditations, where cyclical interoceptive signals processed by the insula from respiratory-related areas such as the diaphragm, chest, and nose, may have an increased effect on cortical activity through increased signal gain [53].

We chose here to focus on the LC due to its crucial role in attentional processes, as we were interested in how the attention might oscillate with the breath. The LC is the primary cortical supply of noradrenaline (NA) to the entire brain via long-range extensive innervation [54,55] and is a global modulator of cortical activation [56]. The LC is involved in arousal, attention, and decision processes [39,57,58,59,60,61], and there is bi-directional connectivity between the LC and frontal attentional areas [62,63,64,65]. The LC plays a particularly important role in attentional state and stability. Optimal focused attention and performance require noradrenaline levels to be tightly regulated within a specific tonic window, and studies have shown that increases and decreases outside of this range are both associated with suboptimal task performance [58]. Pharmacological studies have supported a causal link between modulation of NA and attentional state [66,67]. Crucially, however, the LC has a simultaneous, and important, role as a node in the respiratory system, increasing respiratory activity in response to increases in CO2 [68,69,70,71,72,73]. Removal or chemical inhibition of LC neurons largely abolishes the increased drive to breathe [68,73], highlighting its importance in the respiratory cycle.

Synchronization between LC activity and the breath has been previously observed, with both voxel-based fMRI and pupil dilation (PD; [9]), and because activity in the LC is synchronized with the respiratory cycle, and due to the bi-directional neurophysiological coupling of these systems, we believe the LCs could have a unique position as a bridge between the breath and frontal attentional areas. As blood CO_2_ levels are known to oscillate in phase with respiration [74,75,76], it is reasonable to infer that an induced, possibly subtle, oscillation of electro-cortical activity should be phase-locked to the cycle of respiration.

We thus chose to examine NA release by the LC as a potential mediator between respiration and TBR. While NA release from the LC is difficult to capture directly, due to the LC’s small size and prohibitive location within the brainstem, pupil diameter (PD) has been repeatedly shown to be an easily accessible and highly accurate proxy measure for LC/NA activity in several studies [2,77,78,79,80,81], even down to the scale of single LC neuronal spikes [77]. We examined these potential respiratory-locked fluctuations by measuring respiration, TBR, and PD during quiet eyes-open rest. We assessed synchronization via phase locking value [82], and directional information flow between signal sources via multi-variate Granger causality (MVGC; [83]). Based on our previous findings of respiratory-modulated PD and LC activity, we expected to again observe synchronization of PD, as well as a corresponding oscillation of TBR, in line with the effect of LC/NA activity on attentional state, both synchronized to the respiratory cycle. We also hypothesized a Granger-causal informational exchange bi-directionally between all signal sources, given the established neurophysiological inter-dependence of these systems.

## 2. Methods

### 2.1. Participants

All experimental procedures were approved by the Trinity Research Ethics Committee (SPREC082014-1) and were performed in accordance with the Declaration of Helsinki. Thirteen participants (m(5), *f*(8); age: 22–56) who took part were informed of the testing procedures, made aware that their participation was voluntary, and that their data would be stored anonymously for up to 5 years following the study. Participants gave informed consent to participating by signing a consent form indicating their willingness to participate. Recruiting was carried out through the Trinity College Dublin Department of Psychology subject pool in exchange for course credit, and via advertisements, in which case participants were paid EUR 40 upon completion of testing.

### 2.2. Data Recording

EEG data were recorded from all participants during 8 min of quiet rest. Participants were seated in a chair and were instructed to fixate their gaze on a cross in the center of the screen (~80 cm from the nasion) and think of nothing in particular. Data were recorded from 64 electrodes (standard 10–20 configuration) using a Biosemi Active Two system. Respiratory data were recorded using a SleepSense-1347 respiratory belt fitted with a piezo-electric crystal. Both respiratory and EEG signals were sampled at 256 Hz. Pupil diameter was recorded using an Eyelink 1000 pupillometer, at 1000 Hz, with a fixed head mount to minimize head movement. Pupil data from three participants were excluded from analysis due to excessive blink artefacts and longer periods of eye closing, and EEG data from two participants were excluded due to excessive movement artefacts. In one case both EEG and pupil data for the same participant were discarded. This exclusion criteria resulted in 11 usable EEGs and 10 PD time series.

### 2.3. Data Preparation

All time series were downsampled to 128 Hz. EEG data were re-referenced to the global average, band pass filtered between 1 and 40 Hz, and bad channels were removed. Eyeblink, muscular, and movement artefacts were removed by visual inspection and independent component analysis (EEGLab; [84]). Pupil signals underwent blink removal and were interpolated using a second order polynomial (curve preserving) function. Pupil waveforms were then smoothed with a 250 point Savitzky-Golay moving regression filter to reduce high frequency noise, and normalized to have a mean = 0 and standard deviation = 1. Respiratory waveforms were also smoothed with a 250 point Savitzky-Golay moving regression filter to improve transformation to the phase domain, and similarly normalized. Spectral power values (dB/Hz) were extracted from EEG signals at electrode *Fz* from 1 Hz to 30 Hz (at 0.1 Hz resolution) using a discrete fast Fourier Transform (DFFT; window size = 128 samples, 90% overlap), and theta (3–7 Hz) and beta (13–30 Hz) bands were then extracted. No averaging across electrodes was performed to prevent decreases in signal variance and changes in spectral profile due to requirements for optimization of the MVGC analysis.

### 2.4. Data Analysis

Respiratory waveforms were transformed to their phase angle representations (−π to π) by applying a Fourier-based Hilbert Transform (HT) and extracting angular values. As we were interested in the behaviour of EEG signals locked to the phase of respiration, signals were binned according to their corresponding respiratory phase angle. This was carried out to normalize for differences in respiratory frequency both within, and between participants. Because of this variability, traditional epoching methods (time locking a temporal window to an event of interest) are not optimal if the phase relationship between signals is the subject of interest, which is the present case. The “temporal” epoching process results in an averaging of signal not locked to the respiratory phase angle of interest due to both the divergence of respiratory-signal phase coherence with increasing time from the locking event (e.g., trough of respiration, or π radians), and the varied respiratory frequency between participants. We therefore binned PD and EEG signals within a phase window of interest (π/30 radians, or 6°, here), averaging them for each participant, and then advanced this window in iterative fashion, without overlap, around the complete unit circle resulting in one full respiration of mean signal accurately locked to respiratory phase for each participant.

## 3. Results

**Figure 3:**
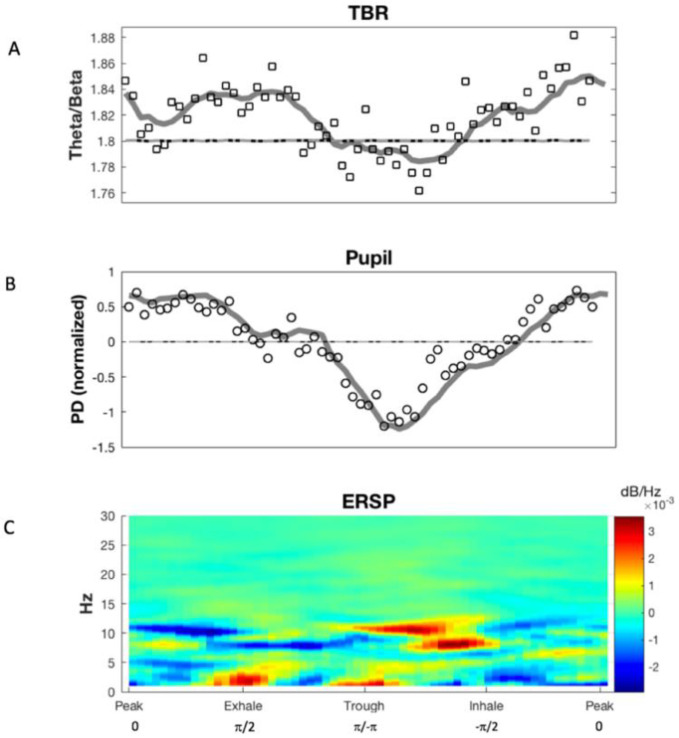
Resulting waveforms of moving phase window method. Bottom phase axis denoted in C by both radians and descriptive terms signifying respiratory phase. Dotted lines (**A**,**B**) show means and 99.73% confidence intervals for surrogate resampling distributions (*n* = 10,000). (**A**). TBR at π/4 radian window size, with 0.25 radian iterative advance (solid black line) and π/30 (unfilled squares; zero overlap) used in statistical analysis. (**B**). PD waveform (from normalized pupil time series) using the same methodology as A (above). Unfilled circles indicate π/30 moving phase window (zero overlap) data points. (**C**). ERSP at π/30 (non-overlapped) resolution, baseline subtracted. Note prominent alpha-respiratory synchronization during early inhalation.

TBR amplitudes showed significant variation across the respiratory cycle according to a repeated measures analysis of variance. This analysis was similar to the methodology of van Son (2019), but with respiratory phase (rather than time) as the within-subject factor, and participant as between-subjects factor (RMANOVA; *F*(10,59) = 1.842, *p* = 0.0002, partial η^2^ = 0.137), and the results indicate a variation in TBR amplitude across the respiratory cycle (Figure 3A) was common across participants. A test for sphericity was inconclusive (Mauchly’s test; *W* = 0, *p* = 1), due to the large number of time points under consideration, and a bootstrap resampling F-test method was employed [85] to test under the assumption on non-sphericity. Resampled distributions were constructed by again using the phase bining procedure, but the bins, rather than being extracted across the true respiratory phase continuum, was randomized with replacement at the participant time series level. An F-test was conducted after each iteration (*n* = 10,000), and a resampling distribution was created. The true test statistic results were significant when compared against this distribution (*F*(10,59) = 1.842, *n* = 10,000, *p* = 0.0004).

The corresponding analysis was also conducted for PD (Figure 3B), and revealed an averaged waveform generally consistent with the TBR oscillation (Figure 3A). This result was also significant according to an RMANOVA (*F*(9,59) = 3.05, *p* = 0.003; partial η^2^ = 0.104), and the test for sphericity was similarly inconclusive (Mauchly’s test; *W* = 0, *p* = 1), and was also subjected to the resampling procedure described above, with a significant result (Figure 3B; *F*(9,59) = 1.627, *n* = 10,000, *p* = < 0.0001).

### 3.1. Phase Synchronization Analysis

**Figure 4:**
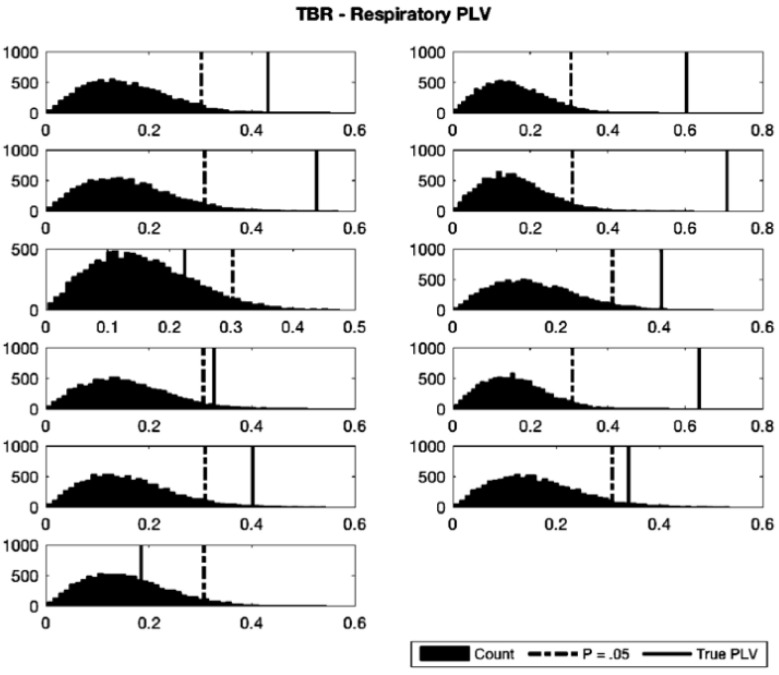
Resampled distributions of TBR-respiratory PLVs for 11 participants. Resampling (*n* = 10,000) was carried out with replacement under the null hypothesis of no relationship between TBR and respiratory phase. Black dotted lines indicate critical values of significance (*p* = 0.05) and solid black lines indicate true participant PLV. Vertical axes show counts of resampled distributions, horizontal axes show computed *p*-values.

**Figure 5:**
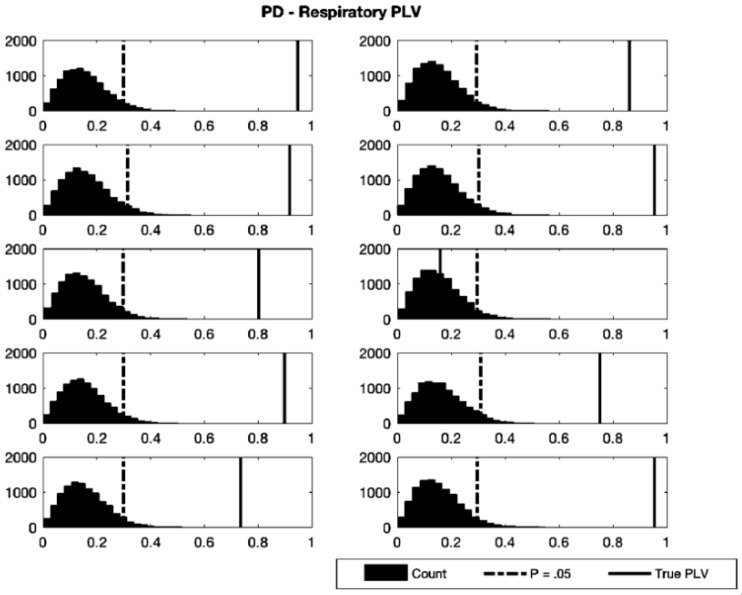
Resampled distributions of PD-respiratory PLVs for 10 participants. Resampling (*n* = 10,000) was carried out with replacement under the null hypothesis of no relationship between PD and respiratory phase. Black dotted lines indicate critical values of significance (*p* = 0.05) and solid black lines indicate true participant PLV. Vertical axes show counts of resampled distributions, horizontal axes show computed *p*-values.

To assess phase synchronization between signals, the averaged TBR and PD waveforms for each participant were Hilbert-transformed to their angular phase representations, and the phase locking values (PLVs) with respiration were calculated. To confirm that the observed synchronization was not due to an artefact produced by the phase binning method, resampling (with replacement, *n* = 10,000) was performed by shuffling of respiratory phase angle under the null hypothesis of no relationship between respiratory phase and the signal of interest (pupil or TBR) while applying the binning method to the original time series for each participant, then calculating the PLV for each surrogate waveform against the true respiratory phase continuum. Probability distributions were created following this method, and the *p*-values for the test PLVs were calculated (Figure 4 and Figure 5; Table 1 and Table 2). Nine participants (out of 11) showed significant phase locking of respiration and TBR according to this metric. To assess significance at the group level, Wilson’s Harmonic *p*-value calculation [86] was used. Harmonic Mean p-value ($\overset{ˇ}{Ρ}$) was calculated by the formula Ρˇ=Σi=1LwiΣi=1Lwi/pi, where *L* is the number of *p*-values, *w_i_* is the *i^th^ p*-value weight (here all weights were 1/12), and *p_i_* is the *i^th^ p*-value. This method is similar to, but preferable in this case to Fischer’s method for *p*-value combining as it does not assume independence of the values. The harmonic mean *p*-value across all participants was $\overset{ˇ}{Ρ}=0$.0003, indicating that the group as a whole showed significant phase synchronization of TBR and respiratory signals. Analogous PD-respiratory analysis resulted in 9 of 10 participants showing a significant degree of phase synchronization, with a group harmonic mean *p*-value of $\overset{ˇ}{Ρ}=$< 0.0001.

(1) $$PLV=\frac{1}{N}\;\overset{N}{\underset{j=1}{\sum }}e^{i\Delta \theta_{j}}$$

**Equation (1)** PLV coefficient [82]. *N* is the number of samples in the time series, *e* is the base of the natural logarithm (Euler’s number), *i* is the imaginary operator, and $\Delta \theta_{j}$ is the angular difference in phase between the j^th^ samples in the two signals. *PLV* returns a value [0 < *PLV* < 1], where 0 indicates unsynchronized (random) signals and 1 reflects perfect synchronization.

### 3.2. Multivariate Granger-Causality Analysis

Many neural and physiological systems both send and receive information from each other. Information is constantly flowing in both directions, and the Multivariate Granger-causality analysis (MVGC; [83]) measures information transfer (above and beyond autocorrelation) in all directions between relevant signal sources. MVGC was performed on the un-windowed time series to quantify informational flow between signal the sources of respiration, PD, respiration, and electrode Fz. As stated above, the topic of interest in the MVGC analyses is information flow between sources. We point out that as TBR is not a source, but rather a descriptive mathematical index of certain frequency bands from that source, the original Fz time series was necessarily used. MVGC utilizes autoregressive modelling, and as physiological signals generally express serial dependence, this method is useful to identify a limited form of causality between source signals above and beyond the autocorrelation present in any pair of given signals. This gives both an approximate magnitude *and* directionality to the information flow (Granger-causality). A further advantage of MVGC, compared to traditional Granger-causal methods, is that it is not limited to two signal sources, allowing an assessment of causal flow in all directions—in this case between respiration, PD, and Fz activity simultaneously.

As we were testing causal flow between all three measures, participants whose PD or Fz data were not usable (see Methods) were excluded from MVGC analysis, resulting in a total of 10 participants. Pupil data were corrected for blinks, but left unsmoothed; EEG data were processed as in the previous analysis; and respiratory data were left in their raw form. This was carried out as raw signals are preferable for MVGC, according to the authors of the method, due to the spectral densities that are calculated during the calculation. Model order, maximum lag, and epoch selection were calculated iteratively per individual until residual autocorrelation was minimized (Table 3).

To test the global null hypotheses that there was no information transfer occurring between pairs of time series across all participants, *Fisher’s method* to combine *p*-values was conducted for each column of Table 4. **χ^2^** and *p* values in the lower row indicate that in all cases significant information transfer was occurring at the *α* = 0.0001 level. Because Fisher’s method assumes independence of individual *p*-values, and it could be argued that the samples were possibly *not* independent due to similarity of testing procedure, equipment, location, experimenter, etc., we chose to use a standard correction measure (false discovery rate; FDR) by first correcting the *p*-value for the number of participants (*n* = 10), and then further, for the number of statistical tests conducted (*n* = 6), resulting in the more conservative criterion of *α* = 0.016. All tests survived this correction.

Eighty percent of residuals tested positive negatively for residual autocorrelation (“whiteness”; Table 5), according to a Durbin-Watson test for residual autocorrelation, the same proportion used in the original authors’ example of MVGC analysis. All DW test statistics were between 1.91 < DW < 2.13 except for one participant with higher residual autocorrelation (DW = 1.7753) in the AR(60) model for pupil diameter. None of the DW statistics fell outside of the values recommended by Field [87], and though the MVGC toolbox specifications also suggest the less conservative values 1 < DW < 3 as informal reference indices for autocorrelation cut-offs, attempts were made to still further minimize residual autocorrelation by increasing the AR model order and maximum lag value within computationally feasible limits (as resource requirements increase exponentially with model order and maximum lag value). This was carried out to ensure that information transfer was indeed occurring above and beyond any serial correlation present within the time series.

## 4. Discussion

We have observed concurrent synchronization of both TBR and PD with respiration by employing three analytical methods that provide congruent evidence of signal synchronization of respiration with TBR and PD at both the group and individual levels of analysis. The three analyses, taken together were intended to show three different aspects of the relationship between these signals: (1) they exhibit phase synchronization, (2) the amplitude of this synchronization is significant, and (3) there is information transfer between signal sources which allow this coupled behavior to occur. The RMANOVA results indicate that TBR amplitude changes significantly over the course of respiratory phase, and the PLV analysis shows that the TBR (Figure 3A) and PD oscillations (Figure 3B) are synchronized to the respiratory cycle. The RMANOVA addresses change in mean signal across respiratory phase, while the PLV assesses signal phase relationships between sources, independent of their amplitudes. Findings of connectivity between respiratory, PD and Fz signal sources, using MVGC modeling, indicate informational flow between all signal sources in all directions, above and beyond any autocorrelation present in the signals, suggesting that the synchronization we observed could be due, at least in part, to this information transfer.

We point out that the MCD index for the *LC* *→ Fz* is generally lower overall that the other pathways (Table 4), though still highly significant (*p* = 0.0004) at the group level. Our overarching theoretical model [9] assumes these nodes are all in fact “weakly coupled”, as weak coupling is a necessary condition for changes in stable phase relationships to emerge in a coupled oscillatory system (as opposed to the simpler limit case of entrainment, where state transitions do not occur). Further, the small amount of information being transmitted between the *LC* *→ Fz*, relative to *Resp* *→ Fz*, suggests there are possibly multiple routes of modulation between the breath and the attentional processes indexed by TBR (see Figure 2). It is likely that in the absence of a focused task where attentional switching might be required, cortical activity is likely idling in the default mode network active state a large proportion of the time. This choice was intentional on our part, as we wanted to isolate oscillatory respiratory-attentional relationships of the resting state, but due to the lack of switching between task-positive and DMN-dominant states and task-related phasic bursts of LC neurons, the influence of the LC on attentional state in our study is likely understated. Future work will assess these dynamics during cognitive tasks, where phasic and regular attentional switching will increase LC signal to the attentional systems.

In the late 19th and early 20th centuries, the idea of an “attentional wave”, or regular periodic attentional fluctuation, was a subject of interest to psycho-physiologists, and respiration was then posited as an explanatory mechanism due to the frequency similarity between the breath and observed oscillations of attention, and the tendency for the attention to shift during specific phases of the respiratory cycle. In 1898, Winkler [19] observed respiratory changes accompanying changes in attentional state, and found that comparable changes in breathing could be elicited by electrical stimulation of frontal attentional areas in the brain. Around the same time, various changes to respiration that were dependent upon stimulus type were also reported [16]. Lehmann [15] noted that changes in attentional state tended to occur predominantly near the beginning of inspiration, and this was later confirmed by Taylor in 1901 [18]. Lehmann also observed that the appearance and disappearance of stimulus on the edge of the perceptual threshold occurred in phase with respiration, with specific phases more conducive to stimulus appearance and disappearance [88]. Respiratory-attentional fluctuations were noted by other early authors as well [89,90], but no comprehensive theory or neurophysiological mechanism could be suggested at the time, and the subject was largely ignored for the next century. More recent researchers have described similar periodic attentional fluctuations, interpreting them as a “refresh cycle” of attention which hypothetically dilates and contracts an opportunistic window which facilitates attentional reallocation by biasing the system regularly toward either exploratory or exploitative behaviors in a periodic, or oscillatory, manner. These cycles express a frequency somewhat greater than 0.1 Hz [91,92,93]. This cycle is hypothesized to have a physiological origin, but no definitive mechanism has thus far been proposed. A similar idea has recently been well-articulated by Sripada as the “oscillatory model” of exploratory/exploitative thought [94]. We believe our findings suggest one possible explanation by suggesting a coupled network, involving the breath, the LC, and frontal midline cortex. Specifically, the fluctuations of TBR and PD may indicate a continual underlying attentional oscillation that is phase locked to the breath, and of the appropriate magnitude of the proposed “attentional refresh cycle”. It should follow from this that respiratory-induced changes in tonic LC activity and cortical NA release should elicit a continual oscillation in signal gain and general cortical arousal that could alternately dilate and constrict the attentional focus upon an attended sensory object. These subtle changes in the primacy of the object of attentional focus, the salience of “background” stimuli (both internal and external), as well as LC input to reward areas in the frontal cortex (OFA; [57]), could bias the attentional system to facilitate such a decoupling of attention.

As mentioned earlier, recent findings have shown that during a breath monitoring task, EEG and fMRI correlates of cognitive control co-vary across mind wandering (MW) events, MW awareness, and the return to focus on the breath. Our own previous research has found respiratory phase-synchronized oscillation of LC (NA) activity, and a corresponding behavioral index of attentional control that was increased at specific portions of the respiratory cycle. Following from this we chose to examine one particular aspect of the breath monitoring task, the breath itself, for covariance with TBR. Our findings confirm that TBR does indeed co-vary with respiration, as does PD. Because LC/NA activity is involved in switching between focused attentional and MW states [80], and its activity is modulated by the breathing cycle, it follows that the respiratory-induced oscillation of LC activity could play a role in an underlying continual cycling of attentional control state; that is, the breath itself could be a contributor to the temporal dynamics of attentional shifting. This idea also fits very well with the early psychophysiological observations described above, where nonlinear perceptual and attentional shifts wax and wane with the respiratory cycle.

It is worth pointing out that there is a noticeable increase in respiratory-locked alpha at the point of lowest TBR (Figure 3C). As previous research has suggested that alpha frequencies are associated with cognitive inhibitory [95,96,97,98,99] and physiological arousal processes [100,101], this could signal some type of attentional braking or sudden increase inattentiveness and arousal during early to mid-inhalation, and this alpha oscillation could have relevance in the context of the oscillation of TBR. This requires further investigation, but given the well-documented relationship between arousal and attention [102] the idea seems plausible. The apparent inverse correlation between alpha and delta frequencies would also support this. This possibility might be clarified by directly assessing arousal via skin conductivity or another suitable co-registration technique.

It is well established that cognitive performance exhibits a non-linear relationship with arousal, where performance declines with either too much or too little [103]. Likewise, arousal is also thought to generally co-vary with attentional state [104,105,106] with low arousal accompanying a drowsy, inattentive state, while higher levels are associated with distractibility. This idea is expressed well by the Adaptive Gain theory [57], which adapts the Yerkes-Dodson curve to the more specific case of LC activity and attentional state. The LC has a neuromodulatory effect on cortical activation, thus a respiratory-induced fluctuation of that arousal could, according to both the specific case of the adaptive gain theory, and the more general case of the Yerkes-Dodson curve, have corresponding attentional and performance consequences, all other things being equal. Furthermore, PD is also known to reflect arousal state [107,108,109], and as we have observed a reliable synchronization of PD with respiration, this strongly suggests an arousal-related component fluctuating with the respiratory cycle. It seems reasonable therefore, to interpret the respiratory-locked oscillation of TBR, generally thought of as an attentional index, at least partly in terms of an underlying fluctuation of arousal in the present context. Overall, therefore, we interpret our results as indicative of a subtle but continual underlying oscillation of attention between bottom-up sub-cortical (possibly chemosensitive- and/or arousal-driven) attention and top-down goal-directed processes.

We believe these findings open an avenue of study on electrophysiological effects of breath focused practices such as pranayama, mindfulness of breathing, and other modern types of “breath-work”. We have observed an attentionally relevant index oscillating in phase with the breath, in a way seemingly commensurate with claims of ancient yogic traditions which posit a direct relationship between the breath and attention. These traditions also teach that by conscious regulation and observation of the breath one can still the fluctuations of consciousness and attention. While anecdotal evidence indicates that this is the case, an empirical method to quantify changes to the respiratory-PD-TBR oscillation could lead to targeted therapeutic applications, and allow assessment of changes to respiratory-related attentional functioning in an objective way. It is not known to what extent training of this oscillation is possible, or what the attentional effects of this would be, but given the long history of the methods of breath awareness and breath control suggesting that respiratory training results in increased attentional stability, future research might address this idea.

The oscillation of TBR, and LC activity with respiration, when considered alongside the fact that respiration tends to become entrained to stimulus presentation [9,110] suggests that differences in task performance could be related to variability in the respiratory-stimulus entrainment of participants. This has indeed been suggested by our previous work, and study designers might take this into consideration during the design process. For researchers wishing to exclude this respiratory effect from their experimental designs, it may be useful to utilize interstimulus intervals (ISI) outside of respiratory frequency, or to use adequate ISI randomization to avoid entrainment.

CO_2_-induced changes in vascular flow in cerebral arteries have been shown to alter fMRI estimations of activity, particularly in functional connectivity studies of the DMN. The suggested solution has been, in a manner similar to RETROICOR [111], to regress out signal changes due to respiratory rate and volumetric variation [112]. According to our present and previous findings, however, the possibility of genuine and important CO_2_-induced changes in cortical activity being regressed out from the data by this particular method deserves serious consideration. The presently observed synchronized changes in respiration, PD and TBR, a measure known to index functional connectivity of attentionally relevant subsystems, suggests that not all of the respiratory-related changes in fMRI functional connectivity studies are due to vascular changes resulting from blood gasses, but rather, a portion of them could be genuine changes in neural activity due not only to the chemosensitivity of the LC, but the other respiratory-related modulators of cortical activity outlined earlier in this paper as well. The implications of this potentially extend to more commonly used forms of respiratory correction procedures like RETROICOR as well, as the regression of instantaneous respiratory effects from fMRI time series might remove genuine signal changes resulting from a small number of other respiratory-cortical modulators (see Introduction), which signify meaningful information about very real changes in brain dynamics. This is a complex, and potentially weighty subject which obviously can not be addressed fully within the scope of this paper, but we believe is deserving of in-depth examination, in line with previous similar suggestions [113].

Finally, as we have outlined previously, we suggest that this respiratory-locked attentional oscillation may facilitate transitions in both directions between mind wandering and focused attentional states. While the utility of an attentional oscillation has obvious benefits for an organism with limited attentional resources, as it allows continual opportunistic cycling between task-focused behaviors and environmental (internal and external) scanning, unintended lapses of attention can also have negative consequences. If attentional state is conceived as a stable attractor state subject to phase state transitions, certain points in the attentional oscillation could provide points of attractor instability during which these transitions become more likely, similar to the quasiperiodic dynamics resulting from phase transitions at points of instability in the classic Lorenz attractor [114]. It would follow from this that if the respiratory-attentional oscillation is malleable, and admits of training through breath-focused practice, that these points might be attenuated, for example by increased frontal-LC white matter connectivity or decreasing CO2 sensitivity in the LC, leading to increased attentional stability, and decreased probability of phase transitions. This idea is addressed, and implied, by our dynamical model of respiration and attention [9]. Training of respiration and attention could thus stabilize this oscillation of attentional processes, and bias their phase-state transitions from being physiologically driven and unconscious to top-down intentional acts. This possibility could be addressed by randomized controlled studies utilizing breathing techniques such as breath-focused meditation, pranayama and other forms of breath control as a treatment and assessing changes in PD and oscillatory EEG dynamics, and physiological changes including CO2-sensitivity, vagal tone, LC volume, and frontal-LC white matter connectivity.

## 5. Study Limitations

The present study lacks a behavioural measure of attention. This was carried out to observe the natural fluctuations of cortical dynamics in the context of the breath without task-related perturbations. It is important to replicate these results with the inclusion of thought probes to track MW events to determine if the fluctuation of TBR we have observed has meaningful behavioral correlates. Nevertheless, the current findings based on natural fluctuations in the resting state, and unadulterated by interruptions of thought probes, provides an important first step to examine these relationships prior to the addition of cognitive manipulations to test the fuller implication of respiratory-attention coupling.

There was significant variation in respiratory frequency, both within and between participants. While this was addressed to an extent by using a moving phase window to normalize respiratory frequency, an important fact to consider is that blood transport time from the lungs to the brainstem is a relatively fixed value while respiratory frequency is free to vary. It is possible that the difference between these two values could cause a phase shift in the synchronization of LC tonic fluctuation from blood gas levels and the respiratory cycle in certain cases. If possible, this issue should also be addressed by future research.

The pupillometric data were collected using a fixed head mount to minimize head movement during data collection. It is not possible to know if this was completely effective, as subtle respiratory-induced head movement may contribute to measured changes in PD. In our previous study, however, pupil data were collected using head-mounted goggles while participants were laying in an MRI scanner, and pupil synchronization with respiration was observed. In that case, because the goggles do not allow for changes in pupil-camera distance, head movement can be excluded as a possible explanation for the synchronization. While this does not exclude the possibility of subtle head movement contributing to PD measurements in the present study, it does suggest otherwise. Still, we recommend that future research utilizing pupillometric measures employ a goggle design headset where possible to exclude this alternate explanation. We believe it would be beneficial to do this in most cases given the apparent natural tendency for respiration to synchronize with stimulus presentation, or as a minimum, in cases where respiration is not a subject of study, to high pass filter the pupil time series above respiratory frequency to eliminate respiratory-induced signal variation, and to employ inter-stimulus intervals outside of the prevailing respiratory frequency to discourage entrainment effects.

The mechanical effect of respiration on EEG electrodes similarly require consideration. Small respiratory-induced movements could have a contaminating ballistographic effect on the EEG signal at respiratory frequency. We believe however, that aside from the filtering of all frequency components below 1 Hz, the frequency bands of interest (theta/beta) were well outside of respiratory frequency range, so effectively this alternate hypothesis can be excluded. Further, we believe the TBR measure excludes any possible global respiratory artefact as the theta and beta signal components are dissociated by the ratio nature of the TBR index. Still, it may be argued that perhaps there is an electrical skin conductivity or resistance change (such as that observed with galvanic skin response) related to respiratory phase within a particular frequency band above 1 Hz. If this were the case, the skin conductivity oscillation should be approximately uniform across the scalp, and because the present EEG data were referenced to the global average, an artefact of this type should not be present.

Finally, the sample size used in this study was relatively small, and the time window for data collection was also relatively short (~110 breaths per participant). The high levels of significance observed in the analyses within these constraints, however, suggest that the underlying synchronization effect of these signals is relatively strong. Regardless, this effect should be replicated with larger groups, possibly as a baseline condition for an experimental manipulation (see Future Research Directions).

## 6. Conclusions

Both TBR and PD show synchronization with the respiratory cycle, and the signal sources of respiration, frontal midline (Fz) activity and PD (LC) exhibited information transfer bi-directionally between all nodes. Taken together the evidence presented here suggests that TBR, an EEG marker associated with attentional control state, is synchronized with respiration, and that respiration, LC/NA, and frontal attentional mechanisms constitute a fully coupled system. This strongly implies a continual, perhaps subtle, underlying attentional oscillation concurrent with respiration, and places the ancient idea of a relationship of the breath and the mind in a modern empirical context, elucidating at least some of the possible dynamics that are expressed by such a system. Future research might consider exploring the dynamics of this oscillation during task, such as breath monitoring or breath control, explore the amenability of this oscillation to training by breath-focused practices such as pranayama, breath awareness, as well as more modern “breathwork” methods, such as the “Wim Hof Method” [115,116], assess the contribution of arousal-related processes, and determine if any therapeutic value can be derived from controlling or attenuating the respiratory-locked attentional oscillation in clinical attentional populations such as traumatic brain injury or ADHD in a targeted, controlled and measurable way.

## 7. Future Research Directions

As mentioned earlier, we did not include a behavioural measure of attention or mind wandering in the present study in order to first observe and describe the natural, unadulterated fluctuations of TBR in the context of the breath. However, we are currently collecting pilot data to examine whether attentional bias occurs across the respiratory cycle in the context of self-caught mind wandering during breath monitoring.

An interesting avenue of study would be to examine the dynamics and magnitude of the TBR and PD relationship in varied populations, such as advanced meditators/breath-workers and attentionally compromised populations including stroke or brain injury patients as well as in conditions such as ADHD. We would expect that the strength of the synchronization might be attenuated in the former populations, and exaggerated in the latter, reflecting differences in top-down control over physiological influences. The pattern of Granger-causal relationships may also prove to differ across these groups due to changes in information transfer between signal sources.

Therapeutic uses of breath focused practices should also be considered. Disorders of anxiety and depression may be particularly benefited by self-modulation of LC/NA and frontal-attentional dynamics with the breath. Given the known involvement of the LC in stress-resilience and cognitive control, and that individual self-regulation has been successfuly indexed by measures of PD [117,118,119,120,121], this possibility and the dynamics that accompany the observed changes, should be explored. Application of breath-focused practices to changes in brain plasticity and neurogenesis might also be a fruitful field of study, as both synaptic remodeling [122] and brain-derived neurotrophic factor (BDNF), particularly in the hippocampus [123,124,125], are modulated by LC activity and NA signaling pathways. It seems plausible that by controlling the breath, NA levels could be optimized to facilitate the formation of neuronal connectivity, and encourage BDNF expression and neurogenesis, given our findings of NA-respiratory coupling.

## Figures and Tables

**Table 1:** *p*-values of participant TBR-Respiratory PLVs against resampled (with replacement) distributions (*N* = 10,000) under the null hypothesis that respiratory phase, which was randomized during resampling, bore no relationship to TBR. Harmonic Mean *p*-value ($\overset{ˇ}{Ρ}$ ) was calculated by the formula |Ρˇ= Σi=1LwiΣi=1Lwi/pi|, where *L* is the number of *p*-values, *w_i_* is the *i^th^ p*-value weight (here all weights were 1/12), and *p_i_* is the *i^th^ p*-value.

Participant	Test PLV	Critical PLV	*p*-Value
1	0.500	0.294	0.0001
2	0.568	0.295	<0.0001
3	0.523	0.296	0.0002
4	0.644	0.300	<0.0001
5	0.239	0.297	0.147
6	0.400	0.297	0.005
7	0.291	0.295	0.046
8	0.588	0.304	<0.0001
9	0.390	0.302	0.005
10	0.103	0.295	0.036
11	0.079	0.296	0.371
			$\overset{ˇ}{Ρ}=\;$0.0003

**Table 2:** Computed *p*-values of participant PD-Respiratory *PLV* against resampled (with replacement) distributions (*n* = 10,000) under the null hypothesis that respiratory phase, which was randomized during resampling, bore no relationship to PD. Harmonic Mean p-value ($\overset{ˇ}{Ρ}$ ) was calculated by the formula. Ρˇ= Σi=1LwiΣi=1Lwi/pi, where *L* is the number of *p*-values, *w_i_* is the *i^th^ p*-value weight (here all weights were 1/12), and *p_i_* is the *i^th^ p*-value.

Participant	Test PLV	Critical PLV	*p*-Value
1	0.948	0.296	<0.0001
2	0.862	0.295	<0.0001
3	0.917	0.305	<0.0001
4	0.955	0.302	<0.0001
5	0.803	0.299	<0.0001
6	0.160	0.297	0.414
7	0.899	0.298	<0.0001
8	0.753	0.306	<0.0001
9	0.736	0.303	<0.0001
10	0.955	0.297	<0.0001
			$\overset{ˇ}{Ρ}=\;$< 0.0001

**Table 3:** Summary of individual model attributes. Model Order (number of autoregressive terms); Autocorrelation Lags (maximum sample lags used in calculation); Epochs (to remedy non-stationarity assumptions in two time series, data was epoched, standardized, and linearly detrended); Mean MCD (mean causal density) MCD values mathematically allow for averaging, hence values were averaged across individual MCDs for each paired time-series comparison; and Harmonic mean p-values tested against FDR-corrected α_adj_ = 0.0292. Grand Mean MCD Harmonic Mean *p*-value tested against FDR-adjusted α_adj_ = 0.0275.

	Model Order	A.C. Lags	Epochs	Mean MCD	Harmonic Mean (*p*-Value)
P1	17	7052	1	0.0002	0.49
P2	63	20,000	1	0.0090	0.00020
P3	50	10,000	41	0.0023	0.00015
P4	15	3144	20	0.0061	0.00012
P5	17	10,000	1	0.0480	0.00005
P6	12	10,000	1	0.0093	0.00020
P7	40	10,000	1	0.0010	0.00055
P8	40	10,000	1	0.0017	0.00020
P9	60	20,000	1	0.0090	0.00030
P10	40	10,000	1	0.0109	0.00028
Grand Mean				0.0097	0.00017

**Table 4:** Summary of MVGC analysis. Each cell contains the MCD (above) and associated *p*-value (below in parentheses). For participant and mean MCDs, an asterisk (*) indicates significance after Bonferroni correction for multiple tests. Mean MCD was also calculated across participants for each granger-causal paired relationship, indicating the general relative magnitude of the causality. The bottom row shows Fisher’s χ^2^ and corresponding *p*-value for the null hypothesis of zero information transfer occurring across the group, as a whole, compared against adjusted α = 0.016.

	Resp Pupil	Resp Fz	Pupil Resp	Pupil Fz	Fz Resp	Fz Pupil
P1	0.00021 (0.61)	0.00027 (0.32)	0.00025 (0.43)	0.00023 (0.51)	0.00018 (0.74)	0.00022 (0.54)
P2	0.0017 (<0.0001) *	0.0016 (<0.0001) *	0.048 (<0.0001) *	0.0009 (0.27)	0.0011 (0.036)	0.0007 (0.79)
P3	0.0009 (0.23)	0.0018 (<0.0001) *	0.006 (<0.0001) *	0.0009 (0.28)	0.0024 (<0.0001) *	0.0016 (0.0001) *
P4	0.0005 (0.01) *	0.0065 (<0.0001) *	0.0019 (<0.0001) *	0.0245 (<0.0001) *	0.0022 (<0.0001) *	0.0010 (<0.0001) *
P5	0.0005 (0.001) *	0.0002 (0.41)	<0.0001 (>0.99)	0.0003 (0.08)	0.2870 (<0.00001) *	0.0005 (<0.0001) *
P6	0.0003 (0.03)	0.0112 (<0.0001) *	0.0062 (<0.0001) *	0.0003 (0.03)	0.0373 (<0.0001) *	0.0004 (0.004) *
P7	0.0005 (0.95)	0.0013 (0.001) *	0.0006 (0.69)	0.0007 (0.45)	0.002 (<0.0001) *	0.001 (0.08)
P8	0.0005 (0.44)	0.0017 (<0.0001) *	0.0015 (<0.0001) *	0.0005 (0.33)	0.0059 (<0.0001) *	0.0005 (0.42)
P9	0.0030 (<0.0001) *	0.0008 (0.098)	0.0483 (<0.0001) *	0.0002 (>0.99)	0.0007 (0.24)	0.0009 (0.01) *
P10	0.0011 (<0.001) *	0.0028 (<0.0001) *	0.0008 (0.06)	0.0005 (0.66)	0.0590 (<0.0001) *	0.0010 (0.002) *
Mean MCD	0.00106	0.002817	0.011365	0.002903	0.039778	0.000782
Fisher’s χ^2^ (p-value)	86.3 (<0.0001) *	133.05 (<0.0001) *	118.60 (<0.0001) *	41.64 (0.0031) *	143.65 (<0.0001) *	96.43 (<0.0001) *

**Table 5:** Durbin-Watson results for whiteness of residuals. Values in bold exceeded DW critical values, but were within tolerances suggested by the original authors of the MVGC method]. All values exceeding critical DW values (bold) were however within the range 1.91 < DW < 2.1, with the exception of a single participant’s (*) pupil model residuals (DW = 1.78). In line with the tolerances used in the original study, 80% of residuals showed no significant autocorrelation, indicating the autoregressive models were overall sufficient to account for the autocorrelation present in the time series.

	Resp	Fz	Pupil
P1	2.0001 (0.99)	2.0040 (0.60)	2.0040 (0.18)
P2	1.9979 (0.77)	2.0017 (0.82)	1.9797 (<0.01)
P3	2.0037 (0.65)	2.0064 (0.43)	1.9965 (0.66)
P4	1.9830 (0.05)	1.9244 (<0.01)	2.0004 (0.97)
P5	1.9778 (<0.01)	1.9999 (0.98)	2.0015 (0.82)
P6	1.9881 (0.10)	1.9997 (0.96)	1.9306 (<0.01)
P7	2.0197 (0.0213)	2.0028 (0.75)	2.0017 (0.85)
P8	1.9961 (0.57)	2.0007 (0.92)	1.9120 (< 0.01)
P9	2.0017 (0.80)	2.0005 (0.95)	1.7753 * (<0.01)
P10	2.0128 (0.09)	1.9996 (0.96)	1.9997 (0.96)

## Data Availability

Data will be made available upon reasonable request to the corresponding author.
